# Supplementary material for: The dynamics of explore-exploit decisions suggest a threshold mechanism for reduced random exploration in older adults
Source: PLoS Comput Biol. 2025 Dec 16;21(12):e1012873. doi: 10.1371/journal.pcbi.1012873 (PMC12742802; doi:10.1371/journal.pcbi.1012873)
Supplement: S1 Text — Table A. Cognitive abilities and tests used for their assessment. If the test was part of a composite assessment, then the composite assessment is in italics. Abbreviations: WAIS IV: Wechsler Adult Intelligence Scale IV, RAVLT: Rey Auditory Verbal Learning test, NAART: North American Adult Reading Test, MINT: Multilingual Naming Task. Table B. Comparison of neuropsychological scores for old and young participants (excluding those who failed the MoCA). Statistics presented are means with lower and upper quartiles in parentheses. P-values correspond to Wilcoxon rank-sum test between the two groups. Table C. Pearson correlation coefficients (r) and significance values (p) between simulated and fit DDM parameters. Table D. Softmax temperatures. Older adult has the average SNR reward and threshold for older adults in this study. Older adult with younger adult threshold has average older adult SNR reward and average younger adult threshold. The increase in temperature in both horizon conditions suggests an increase in noise and so a likely decrease in accuracy. (DOCX) [file pcbi.1012873.s001.docx]

Supplementary Material

# Neuropsychological Testing

The Alzheimer’s Coordinating Center Uniform Data Set 3.0 Battery was administered to participants. This starts with a general cognitive screener, the Montreal Cognitive Assessment (MOCA) [1] and is followed by the Logical Memory Test, Digit Span Forward and Backward, Category Fluency (animals and vegetables), Letter Fluency (both ‘F’ and ‘L’ words) Trail Making A & B, Phonemic Fluency [2], Benson Complex Figure Copy, and Multilingual Naming Task (MINT[3]). Further cognitive tests included the Rey Auditory Verbal Learning test (RAVLT), in which a list of 15 nouns are presented verbally 5 times, to be recalled after each presentation. An interference word list is then presented and asked to be recalled, before immediate and 30-minute delayed free recall of the original list [4]. Selected subtests of the Wechsler Adult Intelligence Scale IV (WAIS IV) were also administered (Letter Number Sequencing, Block Design, Coding, Symbol Search, Vocabulary) [5], in addition to the North American Adult Reading Test (NAART[6]), and Stroop task [7].

Neuropsychological Performance

Neuropsychiatric tests were combined into six cognitive abilities: Memory, Processing Speed, Working Memory, Language Ability, Crystallized Intelligence, and Executive Function. To do this performance on all tasks were z-scored individually across both age groups. We then took the mean z-score for all the tests within a given cognitive ability.

| **Cognitive Ability** | **Tests** |
| --- | --- |
| Processing Speed | Coding, Symbol Search *(both WAIS IV)* |
| Episodic Memory | Craft Story Delayed Recall, Benson Figure Delayed Recall, Delayed recall scores *(RAVLT)* |
| Working Memory | Digit Span Forwards & Backwards, Letter-Number Sequencing *(WAIS IV)*. |
| Crystallized Intelligence | Vocabulary *(WAIS IV)*, NAART |
| Language Fluency | Letter fluency, Category fluency, MINT |
| Executive Function | Trail Making B, Stroop Task, Block Design *(WAIS IV)* |

*Table A: Cognitive abilities and tests used for their assessment. If the test was part of a composite assessment, then the composite assessment is in italics. Abbreviations: WAIS IV: Wechsler Adult Intelligence Scale IV, RAVLT: Rey Auditory Verbal Learning test, NAART: North American Adult Reading Test, MINT: Multilingual Naming Task*

Differences across age groups in these cognitive abilities are shown in Table B. Young adults had significantly better scores in Processing Speed (p<0.001), Episodic Memory (p<0.001), and Executive Functioning (p<0.001). In comparison, older adults had significantly higher scores in Crystallized Intelligence (p<0.001). These results are consistent with the hypothesis that Fluid intelligence decreases with age, whilst Crystallized Intelligence remains constant or increases [8].

| **Cognitive Ability** | **N** | **Older** | **N** | **Younger** | **p-value** |
| --- | --- | --- | --- | --- | --- |
| Processing Speed | 156 | -0.51 (-0.97; -0.0065) | 139 | 0.57 (0.088; 1.10) | <0.001 |
| Episodic Memory | 155 | -0.32 (-0.75; 0.20) | 138 | 0.35 (0.016; 0.78) | <0.001 |
| Working Memory | 155 | -0.060 (-0.57; 0.47) | 134 | 0.062 (-0.45; 0.53) | 0.17 |
| Crystallized Intelligence | 155 | 0.35 (-0.0079; 0.94) | 134 | -0.40 (-1.03; 0.25) | <0.001 |
| Language Fluency | 155 | 0.054 (-0.42; 0.46) | 140 | -0.065 (-0.50; 0.43) | 0.12 |
| Executive Function | 155 | -0.23 (-0.55; 0.031) | 134 | 0.28 (-0.013; 0.55) | <0.001 |

*Table B: Comparison of neuropsychological scores for old and young participants (excluding those who failed the MoCA). Statistics presented are means with lower and upper quartiles in parentheses. P-values correspond to Wilcoxon rank-sum test between the two groups.*

# Distribution of Drift Diffusion Model Parameters

To show the distribution of drift-diffusion model parameters in the Horizon task, S1 Fig shows the same data as Fig 8 but presented as a violin plot. Older adults have a higher threshold (S1 Fig A)(B) and a longer non-decision time than younger adults (S1 Fig B). Older adults have a lower reward signal to noise ratio (SNR) than younger adults in Horizon 1 but not 6 and SNR decreases with Horizon for both groups, but more significantly with younger adults. (Fig S1 Fig C). SNR for information increases with Horizon in both younger and older adults but SNR is lower in older adults under both Horizon conditions (Fig S1 D).

# Parameter Recovery

Parameter recovery was performed using simulated data. Synthetic datasets for 298 participants were generated by simulating choices and response times from the fitted model using the maximum likelihood parameter estimates from the real data. Simulations preserved the original trial structure, including values of reward difference (ΔR), information difference (ΔI), and game lengths. The DDM was integrated using a discrete timestep of Δt=0.001s to compute first-passage times. Simulated datasets were then refit using the same maximum likelihood fitting procedure and parameter constraints as the empirical fits. Recovery accuracy was assessed by computing Pearson correlation coefficients between ground-truth and recovered parameter values across participants. As shown in S2 Fig and table C, there was strong correspondence between simulated and fit parameters. The most important parameters for random exploration (threshold ($c_{0}^{\beta}$) and SNR reward ($c_{R}^{\mu}$)), were near perfect with r>0.91 in all cases.

|  | **Horizon 1** | | **Horizon 6** | |
| --- | --- | --- | --- | --- |
| **Parameter** | **r** | **p** | **r** | **p** |
| $c_{0}^{\mu}$ | 0.823 | 1.03 × 10⁻⁷⁴ | 0.803 | 1.31 × 10⁻⁶⁸ |
| $c_{R}^{\mu}$ | 0.916 | 2.77 × 10⁻¹¹⁹ | 0.926 | 2.45 × 10⁻¹²⁷ |
| $c_{I}^{\mu}$ | 0.905 | 1.03 × 10⁻¹¹¹ | 0.942 | 1.34 × 10⁻¹⁴² |
| $c_{0}^{\beta}$ | 0.938 | 1.44 × 10⁻¹³⁸ | 0.965 | 5.96 × 10⁻¹⁷⁵ |
| $c_{0}^{\alpha}$ | 0.813 | 2.04 × 10⁻⁷¹ | 0.812 | 4.20 × 10⁻⁷¹ |
| $c_{R}^{\alpha}$ | 0.761 | 1.70 × 10⁻⁵⁷ | 0.84 | 1.02 × 10⁻⁸⁰ |
| $c_{I}^{\alpha}$ | 0.747 | 2.40 × 10⁻⁵⁴ | 0.789 | 1.20 × 10⁻⁶⁴ |
| $T_{0}$ | 0.927 | 3.83 × 10⁻¹²⁸ | 0.939 | 1.66 × 10⁻¹³⁹ |

*Table C:* ***Pearson correlation coefficients (r) and significance values (p) between simulated and fit DDM parameters.***

Results of model fitting suggest that both SNR and threshold changes may underlie changes in behavioral variability associated with decreased random exploration in older adults. To understand whether and to what extent this behavioral variability relates to random exploration, we make use of the relationship between the drift-diffusion model (Eq 4) and the logistic choice model (Eq 1). The drift-diffusion model approximates the logistic model with noise given by

| $\sigma=\frac{1}{2\sqrt{\left( 2 \right)}c_{R}^{\mu}c_{0}^{\beta}}$ | *(Eq S1)* |
| --- | --- |

To test the relationship between noise in the logistic model ($\sigma$), SNR reward ($c_{R}^{\mu}$), and threshold ($c_{0}^{\beta}$), we computed the predicted noise parameter from the fit drift-diffusion model parameters (RHS of Eq S1) and compared it to the noise parameter  from the logistic model (LHS Eq S1). These factors were tightly coupled (r>0.54, p<0.0001, S3 Fig, for both horizon conditions (A, unequal, B equal)).

# Softmax Temperature Comparison

To quantify how much a higher threshold in older adults could be compensating for a lower SNR reward, we calculated the softmax temperature for older adults using the average SNR reward and threshold as well as when we replace the threshold with that of younger adults.

Under standard DDM–logistic approximations[9], the probability of choosing the high‐value option is

$$p(high value option)=\frac{1}{1+exp(-2c_{R}^{\mu}c_{0}^{\beta})}$$

Interpreting this as a softmax with temperature τ, we obtain

$$\tau=\frac{1}{2c_{R}^{\mu}c_{0}^{\beta}}$$

We computed group‐mean parameters, calculated the older adult softmax temperature, then the difference between this and when the younger adult threshold is used.

|  | **Softmax temperature** | |
| --- | --- | --- |
|  | **Horizon 1** | **Horizon 6** |
| **Older adult** | 5.168 | 5.788 |
| **Older adult with younger adult threshold** | 6.124 | 7.487 |
| **% change in temp with young threshold** | 18.5% increase | 29.4% increase |

*Table D: Softmax temperatures. Older adult has the average SNR reward and threshold for older adults in this study. Older adult with younger adult threshold has average older adult SNR reward and average younger adult threshold. The increase in temperature in both horizon conditions suggests an increase in noise and so a likely decrease in accuracy.*

There is an increase in temperature with the combination of average older adult SNR reward and younger average threshold. This suggests that older adults would have a decrease in accuracy with the lower threshold of younger adults. This is suggestive that the higher threshold in older adults may act as a compensation for the lower SNR reward.

### Younger and Older Adults Differ in Learning Optimal Strategies Across the Task

Younger adults learned to decrease their threshold while older adults did not. There was a significant Age*Block effect on threshold (F(3,1419.2) = 2.22, p<0.001, η^2^ =0.02, CI[0.00, 0.03], S5 A, S5 E Fig). Younger adults showed a significant decrease in threshold from block 1 to block 4 (t(1419)=4.58, p<0.0001) whilst older adults had a significant increase in threshold from block 1 to Block 2 (t(1419) = -3.24, p= 0.0067). This suggests that younger adults may learn to decrease their threshold while older adults may learn to increase theirs to compensate for their lower SNR reward.

Non-decision time decreased in both younger and older adults at a similar block-rate. There was a significant effect of Block (F(3,1419) = 20.21, p<0.0001, η^2^ =0.04, CI[0.02, 0.06], S5 B, S5 F Fig) but no interaction between block and either age or horizon.

Younger adults seem to learn to increase their SNR reward in Horizon 1. There was a significant effect of Block (F(3,1419.40) = 4.79, p=0.025, η^2^ <0.01, CI[0.00, 0.02], S5 C, S5 G Fig), although no significant interaction of Block with Age or Horizon. However, there appears to be a significant increase in SNR reward from block 1 to block 3 (t(1419) = -3.07, p=0.012) which continues in block 4 (t(1419) = -2.72, p=0.024). This is not seen in older adults, suggesting that the overall higher SNR reward in the whole task in horizon 1 in younger adults, is primarily driven by blocks 3 and 4.

SNR information was relatively stable across blocks with only an interaction of Horizon and Block (F(3,1419.01) = 1.16, p=0.023, η^2^ <0.0024, CI[0.00, 0.01], S5 D, S5 H Fig), in which SNR information was greater in horizon 6 than horizon 1, across all blocks.

# Supplementary References

1. Nasreddine ZS, Phillips NA, Bédirian V, Charbonneau S, Whitehead V, Collin I, et al. The Montreal Cognitive Assessment, MoCA: A Brief Screening Tool For Mild Cognitive Impairment. Journal of the American Geriatrics Society. 2005;53(4):695-9. doi: <https://doi.org/10.1111/j.1532-5415.2005.53221.x>.

2. Weintraub S, Salmon D, Mercaldo N, Ferris S, Graff-Radford NR, Chui H, et al. The Alzheimer's Disease Centers' Uniform Data Set (UDS): The Neuropsychologic Test Battery. Alzheimer Disease & Associated Disorders. 2009;23(2).

3. Gollan TH, Weissberger GH, Runnqvist E, Montoya RI, Cera CM. Self-ratings of spoken language dominance: A Multilingual Naming Test (MINT) and preliminary norms for young and aging Spanish–English bilinguals. Bilingualism: Language and Cognition. 2012;15(3):594-615. Epub 2011/08/01. doi: 10.1017/S1366728911000332.

4. Schmidt M. Rey auditory verbal learning test: A handbook. Los Angeles, CA.: Western Psychological Services; 1996.

5. Wechsler D. Wechsler adult intelligence scale. Archives of Clinical Neuropsychology. 1955.

6. Blair JR, Spreen O. Predicting premorbid IQ: A revision of the national adult reading test. Clinical Neuropsychologist. 1989;3(2):129-36. doi: 10.1080/13854048908403285.

7. Golden CJ, Golden C, Golden C, editors. Stroop Color and Word Test: Manual for Clinical and Experimental Uses1978.

8. Cunningham WR, Clayton V, Overton W. Fluid and Crystallized Intelligence in Young Adulthood and Old Age1. Journal of Gerontology. 1975;30(1):53-5. doi: 10.1093/geronj/30.1.53.

9. Bogacz R, Brown E, Moehlis J, Holmes P, Cohen JD. The physics of optimal decision making: a formal analysis of models of performance in two-alternative forced-choice tasks. Psychological review. 2006;113(4):700.
