# Supplementary figures and images for: The dynamics of explore-exploit decisions suggest a threshold mechanism for reduced random exploration in older adults
Source: PLoS Comput Biol. 2025 Dec 16;21(12):e1012873. doi: 10.1371/journal.pcbi.1012873 (PMC12742802; doi:10.1371/journal.pcbi.1012873)

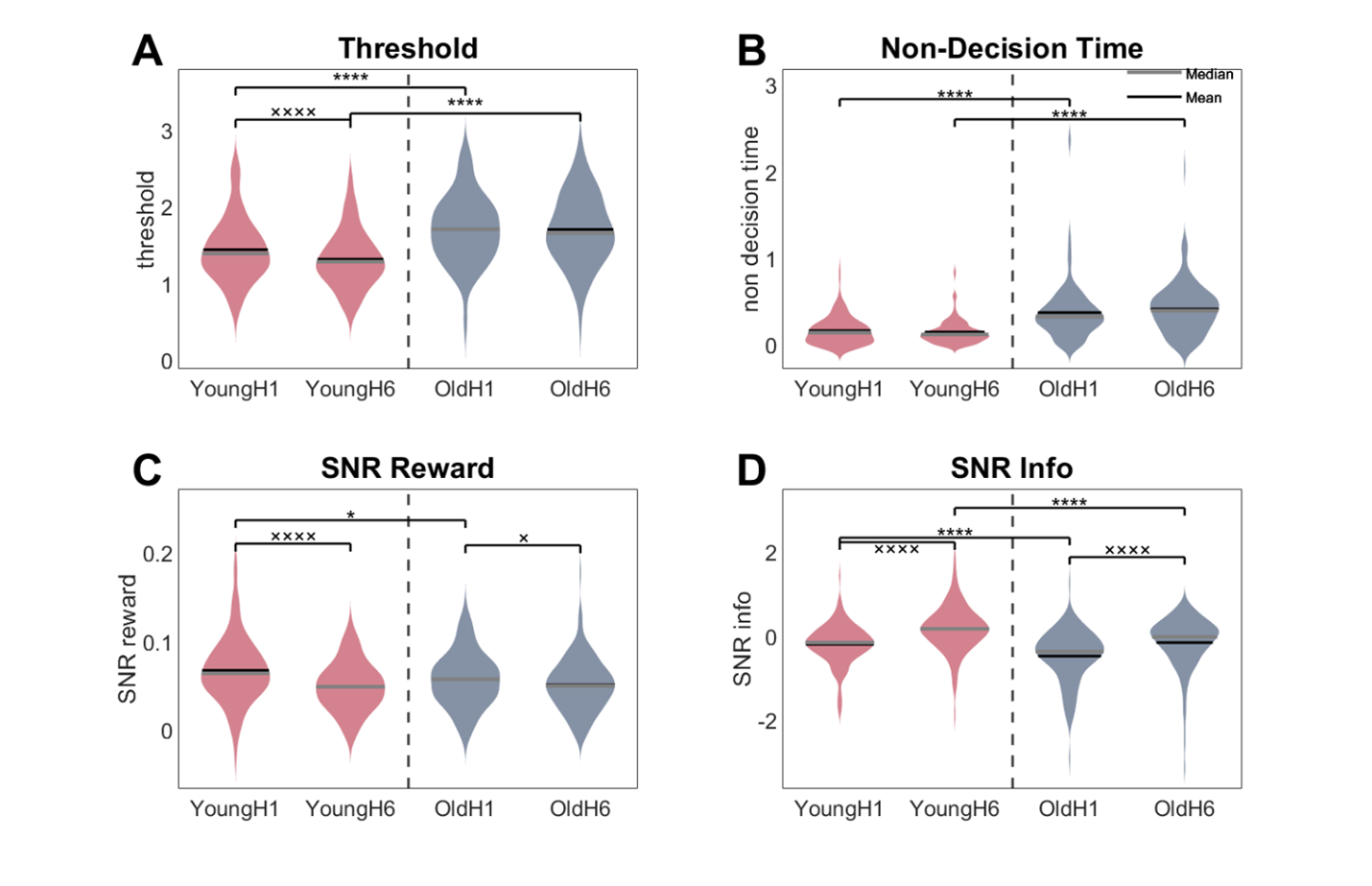

Supplement: S1 Fig — (A) Older adults have a higher threshold than younger adults (B) Older adults have a longer non-decision time than younger adults (C) The signal to noise ratio (SNR) for reward as a function of Horizon. Older adults have a lower reward SNR than younger adults in Horizon 1 but not 6. SNR decreases with Horizon for both groups, but more significantly with younger adults. (D) SNR for information, increases with Horizon in both younger and older adults. The SNR for information is lower in older adults under both Horizon conditions. * p < 0.05, ****p < 0.0001 between age group differences, × p < 0.05, ×××× p < 0.0001 within age group, between horizon differences. (TIF) [file pcbi.1012873.s002.tif]

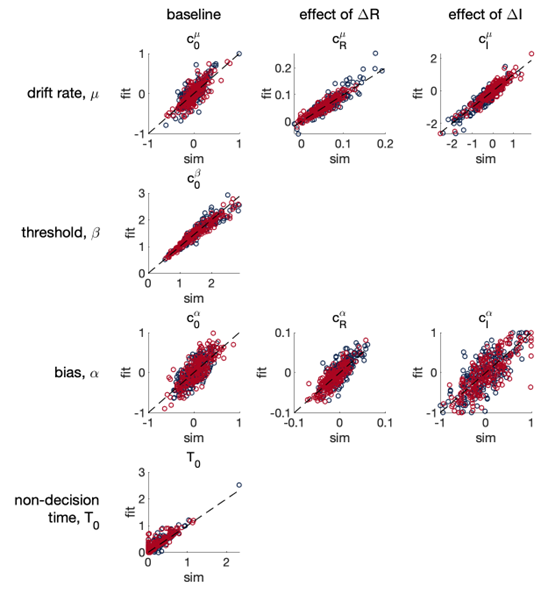

Supplement: S2 Fig — (TIF) [file pcbi.1012873.s003.tif]

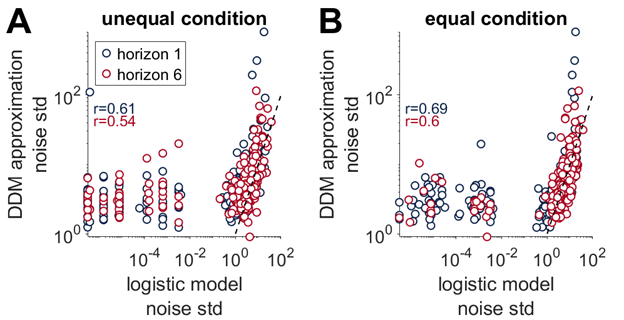

Supplement: S3 Fig — 8 subjects, who had negative drift rate parameters, were excluded from this analysis, leaving 290 participants. (TIF) [file pcbi.1012873.s004.tif]

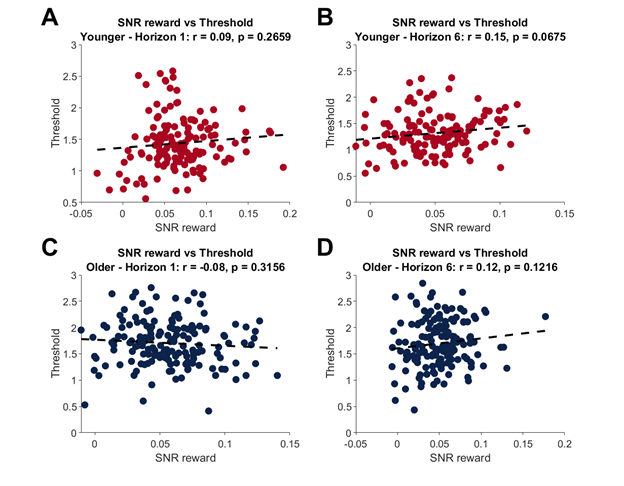

Supplement: S4 Fig — There was no significant correlation between SNR reward and threshold in either younger or older participants in either horizon 1 or 6. SNR: signal to noise ratio. (TIF) [file pcbi.1012873.s005.tif]

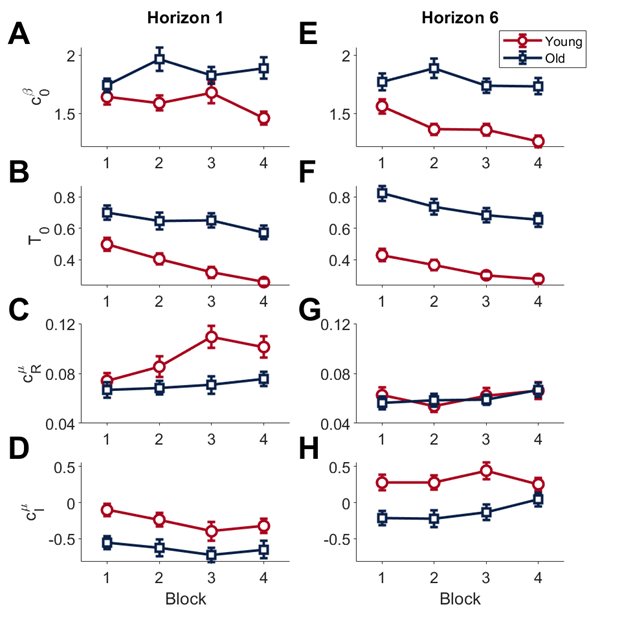

Supplement: S5 Fig — Younger adults also decreased their threshold from block 1 –4, while older adults again did not. This suggests that significant differences in SNR reward and threshold may be driven by learning effects. Horizon 1: A) Threshold B) Non-Decision Time C) SNR reward, D) SNR information. Horizon 6: E) Threshold, F) Non-Decision Time, G) SNR reward, H) SNR information. All error bars are s.e.m. (TIF) [file pcbi.1012873.s006.tif]

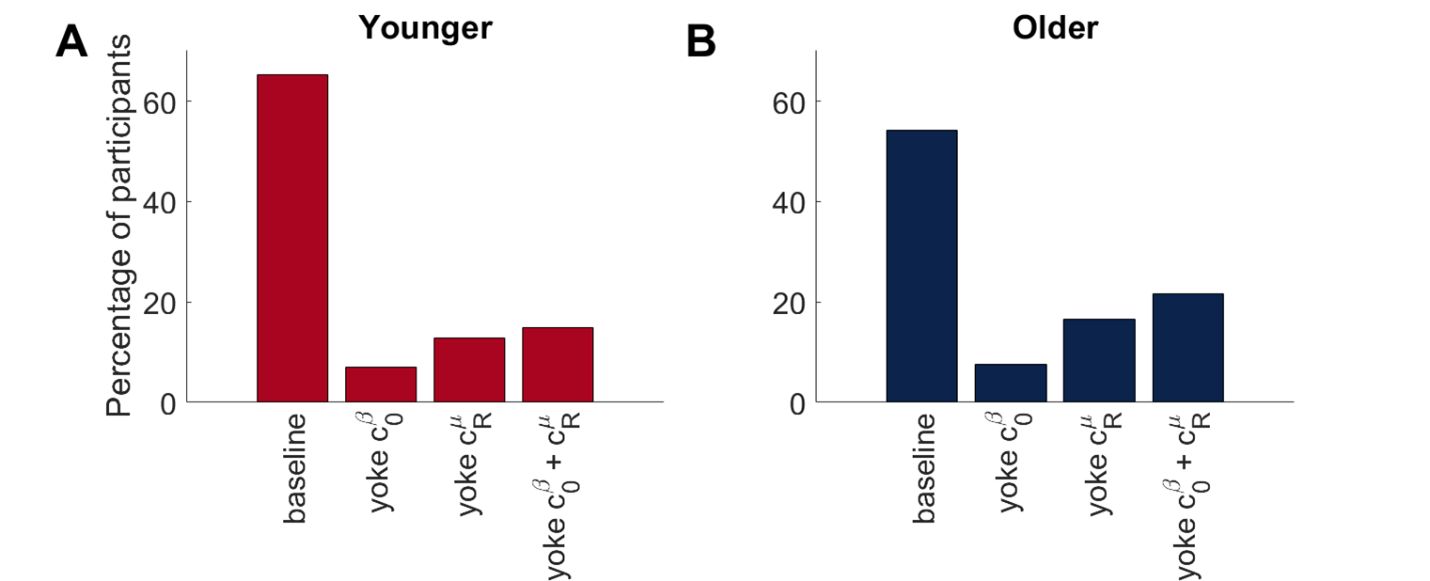

Supplement: S6 Fig — Model comparison was performed using leave-one-out cross validation. The y-axis shows the percentage of participants best fit by each model. The baseline model was the best model for the highest percentage of both younger (A) and older (B) participants. (TIF) [file pcbi.1012873.s007.tif]
