# Supplementary material for: The dynamics of explore-exploit decisions suggest a threshold mechanism for reduced random exploration in older adults
Source: PLoS Comput Biol. 2025 Dec 16;21(12):e1012873. doi: 10.1371/journal.pcbi.1012873 (PMC12742802; doi:10.1371/journal.pcbi.1012873)
Supplement: Attachment — Submitted filename: response_v2.docx [file pcbi.1012873.s009.docx]

6PCOMPBIOL-D-25-00273

The dynamics of explore-exploit decisions suggest a threshold mechanism for reduced random exploration in older adults

PLOS Computational Biology

**RESPONSE TO REVIEWERS**

Reviewer #1: In this paper, Phelps and colleagues use a large data set of younger and older adults in combination with drift-diffusion modeling to generate new insights into aging effects on explore-exploit tradeoffs. Key to the innovation of this study is the use of response times in the Horizons task (a well-established assay of exploratory behavior). Their model-based results show that older adults exhibit reduced random exploration through a combination of lower drift rate and higher threshold.

The paper is well-written and the methods are rigorous. I appreciated the use of an unusually large data set for this kind of study, along with parameter recovery and predictive checks to establish the plausibility of the empirical results. The agreement beteween the data and model shown in Fig. 7 is impressive.

Major comments:

**Point 1.1:** - 8 free parameters fit to each condition for each subject separately seems like a lot, given that there are only at most a few hundred trials per condition (and much fewer in the horizon=1 condition). The parameter recovery results in the supplement are convincing, so I'm not worried that parameter values can be fit accurately. I'm mainly wondering whether have so much flexibility is necessary to fit the data. This is a model comparison question (with a corresponding model recoverability question as well). For example, if you constrain either drift rate or threshold to be the same across conditions, can you still capture the data? Is this more parsimonious model preferred by model comparison?

Thank you for this suggestion. In accordance with your suggestion, we looked at 4 different versions of the model.

Baseline = the current model, yoke $c_{0}^{\beta}$ = threshold the same for both horizons, yoke $c_{R}^{\mu}$ = SNR reward the same for both Horizons, yoke $c_{0}^{\beta}$ and $c_{R}^{\mu}$ = both threshold and SNR reward are yoked. The graph below shows the number of participants best fit with relation to the Bayesian Information Criterion (BIC).

As you can see, yoking both threshold and SNR reward across Horizons, resulted in the lowest BIC value in both younger and older participants. However, there is still a significant proportion of participants who are fit best by the baseline model or yoking only threshold or SNR reward. Thus, to retain flexibility for these participants, we’d like to stick with our current model but have now included this model analysis in the supplementary.

Point 1.2

- In the Introduction, the study is described as 'mechanistic', but I think it's somewhat implausible that the DDM, at least in the way it's used here, is the mechanistic basis for behavior in this task. To apply the DDM, the authors assume that there are relative reward and relative information signals driving the accumulator. This is similar to standard assumptions in applications of DDMs to value-based decision tasks. I'm ready to believe that some sort of evidence accumulation determines choices and RTs in such tasks. What this misses in the Horizon tasks specifically is the fact that one aspect of RTs is (hypothetically) a planning process, which is not modeled here. It could be the case that older adults do less planning, or their planning is more stochastic. This would be closer, in my view, to a truly mechanistic account (such a planning account is implied by the use of dynamic programming to find the optimal solutions in the original Wilson et al. paper, but this point seems to have been lost in the subsequent literature). I don't expect the authors to take on this kind of modeling project, but it's perhaps worth mentioning.

We agree with the reviewer that this is an interesting point and also that it is a substantial amount of work!

Older adults are known to engage differently with the speed-accuracy trade-off than younger adults, such that they are known to take longer to respond and tend to be more accurate – behavioral findings also observed within this paper. However, these results do not tie-in with the idea of more stochastic planning in older adults. In our deep exploration paper (Wilson *et al.* 2020, BioRxiv) we develop a stochastic planning theory of explore-exploit decision making in which the samples are simulations of future choices and outcomes. Within that paper we manipulate the speed-accuracy trade off in an infinite bandits task by increasing the amount of time participants have to respond and a longer time led to an increase in directed exploration and a negatively correlated decrease in random exploration. In the current work, older adults have a longer response time but show a decrease in both directed and random exploration, alongside higher accuracy.

Whilst there are substantial differences between infinite bandits and the Horizon Task, in the supplementary material of that paper, the planning model was shown to also account for behavior in the Horizon Task. Although, unfortunately, as a likelihood-free model, it is much harder to fit. The model in that paper uses an “interrogation” version of the DDM in which integration of evidence stops at a fixed time. We have also (not published) considered a free-response version of the model in which accumulation stops at a particular threshold of evidence, which produces similar results.

We now refer to this work in the paper in the Discussion section on lines 565-570 (approx.).

*“The amount of planning that older adults do during free choice trials or otherwise before their response could also be an adaptation, which may underlie reduced random exploration in comparison to younger adults. We are unable to address this with our current model, but have developed a stochastic planning theory of explore-exploit decision making in which the samples are simulations of future choices and outcomes[42], and future work should also address this possible adaptation in explore-exploit decision-making in older age.”*

Minor comments:

- The paper sometimes refers to 'signal-to-noise ratio' (SNR) and sometimes to 'drift rate'. My understanding that these are the same. I suggest that, to avoid confusion, the authors adopt only one of these terms (I would say 'drift rate' is more standard).

This is a subtle point and we should have described it better. We tried to refer to drift rate as the total drift rate experienced by the model – so

$$\mu= c_{0}^{\mu} + c_{R}^{\mu}\Delta R + c_{I}^{\mu}\Delta I$$

This is slightly different from what we called SNR-reward which is the $c_{R}^{\mu}\Delta R$. We have now clarified our notation referring to $\mu$ as drift rate and $c_{R}^{\mu}\Delta R$ as SNR-reward.

- Please make sure to specify the error bars in all figure captions.

Thanks for bringing this omission to our attention, figure captions all now specify that error bars are s.e.m.

- This is a small point which the authors are welcome to take or leave: currently the paper is not as concise as it potentially could be because all the empirical results are shown once, and then shown again along with the model fits. If the authors wanted, they could compress this to only show them once together.

We were torn with this ourselves. In the end, we decided to stay with the original flow because we wanted to present the model free results

Reviewer #2: The manuscript examined whether exploration-exploitation patterns change as a function of age using the drift-diffusion model framework. Younger adults (ages 20-30) and older adults (ages 60-70) completed the Horizon Task. In this task, participants choose between two bandits one or six times. Before free choice trials, participants are presented with either balanced information (two samples from each option) or unbalanced information (one sample from one option, three from the other). The results indicated that older participants demonstrated less random exploration than younger ones and exhibited slower response times. Analysis of these findings using the drift-diffusion model revealed that older adults had a lower signal-to-noise ratio (SNR) but compensated with a higher decision threshold, resulting in higher accuracy. The researchers interpreted this pattern as an adaptive compensatory mechanism rather than simply reflecting cognitive decline.

The manuscript has several strengths: it addresses an interesting and important research question and the experimental paradigm has empirical foundations for investigating explore-exploit decisions. Additionally, the analysis of both choice behavior and response times enables a deeper examination of the cognitive mechanisms underlying exploration. The use of the drift-diffusion model framework allows to distinguish between competing explanations (reduced SNR vs. increased threshold) for age-related changes in exploration. Furthermore, the findings connect research on exploration-exploitation with cognitive aging. However, in my opinion, the manuscript suffers from conceptual ambiguity and several critical analyses are missing.

Main comments:

1. Conceptual ambiguity – The authors state in the abstract that "Reduced random exploration in aging could therefore be caused either by an increase in signal-to-noise ratio or an increase in decision threshold in older adults". However, I feel that there is a profound conceptual ambiguity in this statement. Within the standard drift-diffusion model framework, participants accumulate evidence based on drift rate, with the "correct" choice determined by the drift direction. In this theoretical framework, there is no conceptual space for "random exploration" – deviations from the drift-favored option are (by definition) errors resulting from noise. However, in the Horizon task a seemingly "suboptimal" choice provides valuable information that may help in subsequent decisions (same as in reinforcement learning frameworks). What appears as "noise" in a single-trial perspective may actually represent information-gathering behavior in a multi-trial context.

The authors attempt to address this by modeling only the first free choice, but this approach does not fully resolve the underlying theoretical tension: when restricted to a single decision point, the DDM inherently treats exploratory deviations as noise-induced errors. Indeed, by employing the DDM in isolation, the authors simplify the complexity of the task and can investigate choice and response time simultaneously. However, an important question that arises is whether the DDM in isolation (applied only to the first choice) is the appropriate theoretical framework for modeling exploration in this task? A possible more theoretically coherent approach would integrate reinforcement learning with the DDM framework.

Couple of points here

Restriction to first trial: A key assumption of the DDM is that responses are made quickly to a punctate stimulus – for example responding left or right in a Flanker task. The critical part here is that the decision process starts when the trial starts. This is a problem for the Horizon Task because the decision could be started before the first free choice trial, during the instructed trials. However, the fast (detection like) responses on the instructed trials, the slow response on the first free choice trial as well as modulation of the first free choice RT by reward suggests that the decision is not started before the first free choice trial. On later trials, however, the decision process is almost certainly more complex involving carry over from previous trials. While this is an interesting target of study, we believe it is beyond the scope of this paper.

DDM framework: Apologies for the confusion on this. In our first paper on the DDM in the Horizon Task (Feng et al. 2021, Scientific Reports), we included a whole section on the link between the DDM and the softmax choice model to show how – under fairly reasonable assumptions – the temperature in a softmax choice rule can be directly related to parameters of the DDM. We now include a brief discussion of results of softmax choice model noise and DDM noise correlation on lines 445 – 450 (approx.) and have included a graph with a fuller description of this relationship in the supplementary (figure S3).

2. Alternative models – The research findings rely exclusively on a single modeling approach. Comparison across multiple model variants would significantly strengthen the robustness of the conclusions. For instance, the authors could compare their full model against: (1) a baseline model that does not differentiate between age groups, (2) reduced models that selectively incorporate information parameters, or (3) more complex integration models including features such as leaky integration, collapsing boundaries or multiplicative relations between ΔR and ΔI. This is important given the central role of the computational modelling in the paper.

Thank you for these suggestions.

Option 1: Here we fit individually so we’re assuming different parameters for each subject, so we are implicitly not differentiating between age groups. Sorry that this wasn’t sufficiently clear.

Option 2: We looked at 4 different versions of the model.

Baseline = the current model, yoke $c_{0}^{\beta}$ = threshold the same for both horizons, yoke $c_{R}^{\mu}$ = SNR reward the same for both Horizons, yoke $c_{0}^{\beta}$ and $c_{R}^{\mu}$ = both threshold and SNR reward are yoked. The graph below shows the number of participants best fit with relation to the Bayesian Information Criterion (BIC).

As you can see, yoking both threshold and SNR reward across Horizons, resulted in the lowest BIC value in both younger and older participants. However, there are still a significant proportion of participants who are fit best by the baseline model or yoking only threshold or SNR reward. Thus, to retain flexibility for these participants, we’d like to stick with our current model but have now included this model analysis in the supplementary.

Option 3: We appreciate this suggestion but believe it to be outside the scope of this current paper.

3. Optimality and compensation – The manuscript would benefit from better defining what constitutes optimal performance in this task. Without this framework, claims about "adaptive compensation" remain speculative. The authors do not quantify the extent to which the threshold increase optimizes performance given the lower SNR. A simulation study demonstrating how different parameter combinations affect cumulative rewards would strengthen their interpretation substantially.

This is an important point that we missed. So, we have now added in reference to optimal performance in the Horizon task as calculated in Wilson et al 2014, under the choice behavior section. Please see lines 276-298. Briefly optimal performance consists of zero decision noise, and 0 information bonus in horizon 1, but an information bonus of 3 in horizon 6. Younger adults are closer to information bonus ideals, but older adults have lower decision noise, which is closer to the zero optimal figure.

As a way to quantify the extent that the threshold increase optimizes performance given the lower SNR in older adults, we have computed the softmax temperature with the average younger adult threshold applied to older adult SNR reward and then looked at how much ‘hotter’ or more random the behavior becomes.

Under standard DDM–logistic approximations (Bogacz et al., 2006), the probability of choosing the high‐value option is

$$p(high value option)=\frac{1}{1+exp(-2c_{R}^{\mu}c_{0}^{\beta})}$$

Interpreting this as a softmax with temperature τ, we obtain

$$\tau=\frac{1}{2c_{R}^{\mu}c_{0}^{\beta}}$$

We computed group‐mean parameters, calculated the older adult softmax temperature, then the difference between this and when the younger adult threshold is used.

|  | **Softmax temp** | |
| --- | --- | --- |
|  | **Horizon 1** | **Horizon 6** |
| **Older adult** | 5.168 | 5.788 |
| **Older adult with younger adult threshold** | 6.124 | 7.487 |
| **% change in temp with young threshold** | 18.5% increase | 29.4% increase |

There is an increase in temperature and so randomness, suggesting a decrease in accuracy with the lower threshold of younger adults. This is suggestive that the higher threshold in older adults may act as a compensation for the lower SNR reward. We now include this table in the supplementary (Table S3) and reference it in our main paper in the results section (lines 459-464).

4. Parameter recovery – Given that the authors' conclusions rest on comparisons between model parameters, it is essential to show robust parameter recovery analysis. While they report recovery results in Suppl. Fig. 1, the details about how the recovery was performed are not specified. Additionally, it would help if the authors add the correlation between the recovered and generated parameters for each parameter. Furthermore, the authors should examine trade-offs between parameters, specifically between the drift related and response boundary parameters. This is important to establish that the results do not stem from statistical artifacts.

We have now added further details for parameter recovery and correlation coefficients, alongside the previous figure in the supplementary.

**Trade‑off analysis**
To address the reviewer’s concern about statistical artifacts between SNR and threshold parameters, we used both the empirical and simulated data to look for any correlations between these parameters.

In the simulated data, we found no correlation between SNR reward and threshold in Horizon 1, but we did find a significant correlation in Horizon 6 between SNR reward and threshold for both younger (r=0.21, p=0.012) and older (r=0.21 p=0.0076) participants.

However, crucially, our empirical Horizon 1 and 6 fits show no such correlation indicating that the dissociation between drift and threshold effects in the real data cannot be attributed to any trade-off artifact of the fitting procedure.

**Simulated Data**

**Empirical Data**

5. Compensation mechanism – The authors propose that older adults use higher decision thresholds to compensate for reduced SNR. However, the compensatory interpretation requires more rigorous evaluation. Does this relationship take place at the individual participant level (correlation between threshold and SNR parameters at the individual level), or is it only observable at the group level? Is this compensatory adjustment a conscious strategic adaptation or an implicit process?

Thank you for this valuable point. When we looked at the correlation between SNR reward and threshold (see empirical figure above), we see that there is no significant correlation between SNR reward and threshold on the individual level. However, this is something worthy of further study, as our task was not set up to look at individual correlations. Furthermore, knowing whether compensatory adjustment was strategic or implicit is hard to distinguish in this task. We have added the correlation graph into the supplementary and mentioned that this potential compensatory adjustment is not on the individual level in the current task.

Minor comments:

6. Theoretical framework – The paper proposes that differences in SNR and decision boundary between age groups, but these observations are insufficiently grounded in theoretical framework on cognitive aging and decision-making processes. Why do older adults exhibit lower SNR? Is it the result of difficulty in processing information or a matter of visual acuity? Was visual acuity controlled for?

This is a good question and one we attempted to answer in the discussion, sorry that this was not as clear as it could be. The main theoretical literature focuses on the idea of older adults being intrinsically noisier – thus reducing the signal to noise ratio. The most common theory for this is reduced neuronal dedifferentiation which we already discuss on page 28. However, a reduction in the signal magnitude is also a possibility and older adults have been shown to have diminished reward magnitude processing in decision making (e.g. Chen et al 2022, Scientific Reports, Dingha et al 2019, Neuroimage). I see that we have missed out talking about reward magnitude and have now included a brief description of this in the discussion.

Thanks for the thoughts on visual acuity. If participants had eyesight issues and wore glasses, they had to have prescription between +3 and -5. This criterion was in place for an MRI session that is not reported here, where we only had lens for the MRI goggles between those prescriptions. We did not explicitly test eyesight in this task, but participants had to be able to read the onscreen instructions sufficiently well to be able to participate. In the future we could include a brief eye check at the beginning of these experiments to control for this potential age-related variable.

7. Parameter distributions – The analysis would benefit from data about parameter distributions across participants rather than relying solely on group means.

We appreciate your opinion, and whilst we would like to stick with our current presentation in the main paper, we have now included this violin plot of parameters in the supplementary.

8. Statistical power – While the sample size is substantial and the study appears adequately powered, the authors do not specify how sample size was determined.

We have added this sentence on sample size under ethics statement.

*The sample was collected as part of grant (R01 AG061888)) in a preregistered clinical trial (NCT05178381). Sample size was determined to provide enough participants (after exclusions) to achieve 80% power for two TMS studies (not reported here).*

9. Learning effects – Do the observed age differences remain stable throughout the task or evolve differently for older versus younger adults as they gain experience with the task structure? Comparing behavior between early and late blocks could reveal differences in how exploration strategies adapt across the experiment.

Thank you for this valuable point, doing your suggested analysis brought us to an exciting finding! As you can see in figure C below, SNR reward increases in block 3 and 4 in younger adults only and only younger adults lower their threshold across blocks. This suggests a significant learning effect only in this age group. We’ve now included this figure in the supplementary.

*Figure S5: Younger adults seem to increase their SNR reward in Horizon 1 during block 3 and 4, while older adults do not. Younger adults also decreased their threshold from block 1 to 4, while older adults again did not. This suggests that significant differences in SNR reward and threshold may be driven by learning effects. Horizon 1: A) Threshold B) Non-Decision Time C) SNR reward, D) SNR information. Horizon 6: E) Threshold, F) Non-Decision Time, G) SNR reward, H) SNR information. All error bars are s.e.m.*

10. Figure 5 notations – The ‘i1’, ‘i2’ notations in Fig. 5 are not defined in the caption.

Thank you for catching that. The meaning of the i1, i2 etc., notations are now included.

Reviewer #3: The manuscript "The dynamics of explore- exploit decisions suggest a threshold mechanism for reduced random exploration in older adults" by Phelps et al. describes a study that was aimed at investigating the origins of changes in explorative behaviour in older adults (65-74 years) compared to younger ones (18-30 years) using a Horizon task and drift diffusion modelling (DDM) to distinguish between directed and random exploration. Previous studies had shown effects of reduced exploration and increased exploitation in older adults. But while changes in random exploitation had been suggested as potential underlying mechanism, the exact mechanism had remained unclear.

Here, using the advantage of DDMs by fitting both choice and reaction times as part of the modelling, the authors could show lower Signal To Noise Ratio and a higher (decision) threshold in the group of older participants. They suggested that the decrease in random exploration shown in older adults might be driven by the elevated decision threshold, which could be a way of compensating for the second finding, namely a reduction in SNR.

This study asks a really interesting research question, that has become more central to the learning and decision-making literature recently with papers such as Findling et al. 2019 and 2021 discussing the different aspects of decision noise. Moreover, the study uses an appropriate task to distinguish between different aspects of explorative behaviour and employs advanced methods to address the research questions posed here. Thus, I would consider the results to be relevant for the scientific community and a valuable and interesting addition to the field of decision science. Overall, I would be in favor of this study being published, given that my concerns and questions are addressed.

Comments to the authors:

Introduction:

p. 6, ll. 93-96. Here, the sentence structure seems to be grammatically incorrect or at least it sounds confusing to me. Hence, I would suggest rewriting this sentence to make it clearer.

Thank you for this suggestion, we have revised the sentence in order to make it clearer.

My next comment is regarding a specific part of the manuscript (p. 8, ll. 133-137). Unfortunately, for a reader who is not very familiar with the Horizon task, this paragraph makes a lot of assumptions and the claims seem somewhat ad-hoc. Consequently, I would suggest rewriting the paragraph and explain in a bit more detail, how they arrive at claims such as ‘This suggests that horizon-dependent directed exploration is similar in younger and older adults.’ or ‘This suggests that horizon dependent random exploration is reduced in older adults.’ that seem to be based on the findings by the study of Mizell et al.

This is a good point, we have added in a description as to how directed and random exploration is calculated, alongside reference to our method figure which helps explain this better (figure 9).

My next concern is about the way the DDM is described in the introduction (p. 8-9, ll. 139-161). While some of what is described here might be considered common knowledge, I would still add the respective citations here. Though I realize that the authors describe the DDM extensively in the method section including the appropriate citations, given the structure of the manuscript with results coming first, this is still helpful.

We agree and have added relevant citations to the text at the requested point.

Results

On p.15 it is unclear what the authors mean by spatial bias in the model, as it is not explicitly stated in this part of the paper, as far as I can see (p.15, ll. 254). I would suggest to add a short description here as well to make this easier for the reader.

Added in that the spatial bias is related to a bias for choosing the left bandit.

p. 18 starting l. 305 the authors say ‘The degree of this reward-response time modulation appears to differ between younger and older adults, so…[…]’ This suggestion comes somewhat out of the blue, as the previous paragraph does not say so and while you probably base this on Fig 6, this is not explicitly stated and therefore confusing. It needs to be made clear, on what this statement is based on, so please consider some rewriting of this paragraph.

Have rephrased and included a reference to the figure.

Methods

In general, given that neurocognitive test scores are available for both samples and the supplement describes a few key differences in cognitive scores between the groups (even though they are in the expected direction when considering age), I would be interested in how the differences between the two groups are related to or might have impacted the findings. Specifically, do the main results in terms of changes in random exploration for the older participants still hold when corrected for neurocognitive differences? Or, as one might speculate that those neurocognitive changes mediate the changes in explorative behaviour, have you assessed the mediating effect of neurocognitive functions on the association between age and exploration (SNR reduction & threshold)? I consider this especially relevant, given that the authors are listing the potential changes in crystalized intelligence in older adults as potential origin of the changes in the discussion (ll. 441-444).

Thank you for this suggestion. We have previously looked at the correlations between neurocognitive scores and directed and random exploration in the Horizon Task in schizophrenia patients (Waltz et al 2020, Comp Psych). There, most neuropsychological faction correlations were with overall performance, overall information seeking and overall behavioral variability, with only verbal learning correlating with random exploration.

We agree that it is important to investigate if neurocognitive differences between younger and older adults mediate the main results reported here. However, understanding the role of neuropsychological factors on directed exploration and overall behavioral measures is also important, but adding them in here would dilute the main message of this current paper. We are currently working on a second paper which will address exactly this point.

I would also suggest to add some more information about the parameters in the DDM section of the paper, for instance the authors could list the 7 parameters again in brackets. This way, every reader will know right away which parameters the authors are referring to here (p. 39, ll. 663-672). Also, I was wondering, why did you choose to fix c instead of any of the other parameters?

Added in at 366 where the results are discussed … As shown in Fig 7, the drift diffusion model provides an excellent fit to the choice and response time data on the first free choice trial. “The free parameters of this model are the 7 coefficients, $c_{j}^{i}\left( i \in\left\{ \mu, \beta, \alpha\right\}; j \in\left\{ 0, R, I \right\} \right)$ and an additional non-decision time $T_{0}$. Each of these parameters are assumed to vary with Horizon, giving 16 free parameters.”

We chose to fix c as 1, because this is standard in the DDM literature (e.g. Bogacz et al 2006 and Ratcliff & McKoon, 2008). We cannot dissociate all three (threshold (β), drift rate (µ) and noise (c)), so in terms of our main aims, it makes most sense to fix noise and focus on threshold and drift rate changes in aging.

Finally, I was wondering whether the authors could check if/ how many of the participants had chance fits when fitting the DDM and then rerun analysis without them to see whether results remain stable. This is an approach often used for reinforcement learning models, though I am not sure, whether this is a common thing done for DDMs.

Thanks for this suggestion. We avoided applying either performance cut-offs or controls for chance fits in the main DDM figure, because we were concerned this could bias our results, because a greater number of older participants are excluded. See table:

|  | **Younger Participants** | **Older Participants** |
| --- | --- | --- |
| **Total in dataset in paper** | **141** | **157** |
| **Exclusions** | | |
| Performance exclusion (accuracy above 50% on trial 6) only | 1 | 3 |
| Chance fit (SNRs below 0.1) only | 17 | 25 |
| Both performance and chance fit | 1 | 0 |
| **Total Excluded** | **19** | **28** |
| **Total after exclusions** | **122** | **129** |

However, we agree it’s important to determine whether the results remain stable. We followed your advice and please see a comparison below.

| **Figure 8: Main Paper** | **After Exclusions** |
| --- | --- |
|  |  |

As you can see there older adults still have a lower SNR than younger adults and a higher threshold that doesn’t change with horizon.

General

Finally, many of the figure captions are extremely short and some of them could benefit from additional, though brief description of what is depicted in the respective panels, i.e. mini summary of the finding. While a lot of this is also described in the main text of the manuscript, figures and figure captions need to be treated as ‘stand-alone’ items and in theory should be comprehensible without the main manuscript. Also, some information is simply missing from the captions, such as in Fig 5. Here, it is unclear what i1-i4 means and the reader has to search the manuscript for this information.

I have updated the figure captions to include a brief summary of the finding in each panel. Thank you for also pointing out the missing information in Figure 5, this has also been amended.
