# Supplementary material for: The dynamics of explore-exploit decisions suggest a threshold mechanism for reduced random exploration in older adults
Source: PLoS Comput Biol. 2025 Dec 16;21(12):e1012873. doi: 10.1371/journal.pcbi.1012873 (PMC12742802; doi:10.1371/journal.pcbi.1012873)
Supplement: Attachment — Submitted filename: 2_Response_to_Reviewers.docx [file pcbi.1012873.s010.docx]

**Response to Reviewers**

***Reviewer #1:*** *The authors have done a good job addressing my comments.*

***Reviewer #2:*** *I thank the authors for addressing my comments.
My main remaining concern is the model comparison (Fig. S6). The results indicate that a model yoking H1 & H6 parameters achieves the lowest BIC (preferred for ~70% of participants), whereas the flexible baseline performs worst (preferred for fewer than 10%). Because DDM parameters are model dependent, parameter differences derived from the statistically inferior baseline model raise overfitting concerns and are not justified as presented.
I believe that instead of burying this analysis in the supplement, the authors should be more transparent about the results, move them to the main text, and reframe and soften claims relying on the flexible specification. Approaches I consider preferable are: 1. Compute parameters via model averaging (based on BIC or AIC weights) and use those parameters for group/horizon comparisons. 2. Compare models using cross-validation and show that the gain (in likelihood terms) for the flexible model is substantial. 3. Model the task differently, for example, a model assuming a starting-point bias might fit better than one assuming a change in drift.
I leave it to the authors and the editor to decide which approach to take.*

Thank you for your further review and helpful suggestions. We agree that leave-one-out cross-validation is a better way to do model comparison. Please see our revised figure below.

*Baseline = the current model, yoke* $c_{0}^{\beta}$ *= threshold the same for both horizons, yoke* $c_{R}^{\mu}$ *= SNR reward the same for both Horizons, yoke* $c_{0}^{\beta}$ *and* $c_{R}^{\mu}$ *= both threshold and SNR reward are yoked.*

Leave-one-out cross-validation showed that our unyoked baseline model had the lowest test loss for the majority of participants. All yoked models are special cases of the more general baseline model, so taking this into account, alongside the cross-validation results, we decided to use our baseline model for analysis.

Our figure legend now reads:

*To determine whether as much flexibility as we have in the current model (baseline) is needed, we conducted model comparison with threshold yoked (yoke* $c_{0}^{\beta}$*), SNR reward (yoke* $c_{R}^{\mu}$*) or both threshold and SNR reward (yoke* $c_{0}^{\beta}$ *+* $c_{R}^{\mu}$*) yoked across Horizons. Model comparison was performed using leave-one-out cross validation. The y-axis shows the percentage of participants best fit by each model. The baseline model was the best model for the highest percentage of both younger (A) and older (B) participants.*

In the text on lines 481-484:

*To determine if a more parsimonious version of our model may be better, we conducted model analysis using leave one out cross validation (Figure S6). Here we yoked SNR reward, threshold or both SNR reward and threshold across horizon conditions. We found that the full baseline model used here, was the best fit for the majority of younger and older participants.*

***Reviewer #3:*** *First of all, all of my comments have been addressed by the authors and the appropriate revisions have been made to the manuscript.

However, I am concerned after having also gone over the issue raised by the other two reviewers regarding the potential need of extending the model space by additional models and the authors‘ response to this. I agree with the concern raised here, even though I had not raised it myself.

I personally find the argument made by the authors of why they eventually chose to stick to the baseline (original model) somewhat arbitrary. They say that they would want to stick to the original model given that quite a number of participants in each sample are best fit by this model or one of the two new models including a yoked threshold or SNR reward and that they want to retain flexibility for those participants.

While it is true that about 55 participants are fit best by one of the other models, with the least number of people fit best using the original baseline model, a very large part of the sample is fit better by the fourth model (from what I can tell based of the figure, more than 80 and 100 participants respectively), and this applies to both the older and younger sample. It does not really make sense to use model comparison if one just picks the model that one originally intended to analyze, independent of what the BIC indicates. Also, even when sticking to the original model and ignoring the results of model comparison, these results just seem to be included in the supplement without any further discussion of why so many participants might have been better fit by another model or what the implication might be for the results. I feel like this is not sufficient here and would suggest additional analysis including the winning model or at least a further discussion of the modelling results including the discrepancy of the analyzed model vs. the winning model.*

Retrospectively, with the suggestion from reviewer 2, we realized that leave one out cross validation is better for analyzing DDMs, so we have now done model comparison with this methodology. Please see below.

*Baseline = the current model, yoke* $c_{0}^{\beta}$ *= threshold the same for both horizons, yoke* $c_{R}^{\mu}$ *= SNR reward the same for both Horizons, yoke* $c_{0}^{\beta}$ *and* $c_{R}^{\mu}$ *= both threshold and SNR reward are yoked.*

Leave one out cross validation showed that our unyoked baseline model had the lowest test loss for the majority of participants. All yoked models are special cases of the more general baseline model, so taking this into account, alongside the cross validation results, we decided to use our baseline model for analysis.
